# “Finding my own identity”: a qualitative metasynthesis of adult anorexia nervosa treatment experiences

**DOI:** 10.1186/s40359-020-00476-4

**Published:** 2020-10-22

**Authors:** Janet E. Conti, Caroline Joyce, Phillipa Hay, Tanya Meade

**Affiliations:** 1 School of Psychology, Western Sydney University, Locked Bag 1797, Penrith, 2751 Australia; 2 School of Medicine, Western Sydney University, Penrith, Australia; 3 Translational Health Research Institute, Western Sydney University, Penrith, Australia

**Keywords:** Anorexia nervosa, Metasynthesis, Qualitative, Adult, Treatment, Therapy, Recovery, Identity, Personal agency

## Abstract

**Background:**

The aim of this metasynthesis was to explore adult anorexia nervosa (AN) treatment experiences, including facilitators and barriers to treatment engagement and ways that questions of identity and personal agency were negotiated in treatment contexts.

**Methods:**

From 14 qualitative studies that met the search criteria, this thematic synthesis analyzed the sensitized concept of identity in the participants’ experiences of AN treatments, including their sense of personal agency, and implications for their recovery. The study was registered with Prospero (ID: CRD42018089259) and is reported according to PRISMA guidelines.

**Results:**

Three meta-themes were generated with the following key findings: grappling with identity, where collaborative and tailored interventions were positively experienced; the quality of the therapeutic relationship, which existed in a recursive relationship; and, rebuilding identity that included therapists standing with the person in recovering a sense of identity outside the anorexic identity. Importantly, interventions that failed to be negotiated with the person were experienced as disempowering however, where a two-way trust existed in the therapeutic relationship, it critically empowered and shaped participants’ sense of identity, and broadened the perception that they were valuable as a person.

**Conclusions:**

There was consensus across the range of treatment contexts that individuals with a lived AN experience preferred treatments where they experienced (1) a sense of personal agency through tailored interventions; and (2) therapists who treated them as a person who, in the face of their struggles, had skills and capacities in the processes of recovering and rebuilding sustainable and preferred identities outside the AN identity.

## Background

### Rationale

There have been major advances in empirical research testing psychological therapies for people with anorexia nervosa (AN) in the past two decades [1, 2]. Nevertheless, evidence is incomplete and there has been insufficient replication of findings [1, 3]. Some treatments may be efficacious at particular time points such as Specialist Supportive Clinical Management (SSCM) delivered by ED specialist practitioners with a focus on nutritional counselling and weight restoration in early stages of treatment [1]. Family based treatment is the leading therapy for young people [4, 5], nevertheless, a Cochrane review of the research evidence [6] reported inconclusive findings as to whether family therapy is more effective in the long term compared to other psychological interventions for adolescent AN. Similarly, there is no therapeutic intervention for adult AN that has consistently resulted in positive treatment outcomes [1, 2].

Complexities inherent in AN are likely a reason for the challenges in evidence-based treatment research. Many people with AN are ambivalent about help-seeking, leading to avoidance and low engagement in treatments, even when AN is potentially life-threatening [7]. The desire to recover from AN may co-exist with significant ambivalence to behavioral change, as evidenced by half of the cases of lifetime prevalence of AN in Finland (2.2% population) being undetectable in the healthcare system [8]. AN has highly egosyntonic features [9] and a potential to run a chronic course with significant impacts on a person’s quality of life [10]. Furthermore, a substantive proportion of people do not engage in specialized AN treatment [8, 11], and for those who do, treatment drop-out rates are notably high [12, 13]. Research into AN treatment interventions has focused on addressing weight and eating behavior change with outcomes measured on these variables and the presence or absence of other psychiatric diagnoses [13]. These may not be the outcomes that are a priority for people with a lived AN experience, particularly those with longstanding struggles where quality of life may be a more important goal [14].

Engaging adults in AN treatment is thus complex, particularly around the competing needs of individual choice, personal agency (or the sense of being able to play an active role in one’s life according to one’s values [15]) and the delivery of mandatory treatment components that prioritize the person’s physical and psychological safety or treatment non-negotiables [16]. Research into AN treatments from the perspective of people with the lived experience can contribute to an in-depth understanding and has potential to inform clinically and developmentally appropriate treatments that align with the person’s needs and values [17]. While the experience of adolescents has been examined in recent metasyntheses [18, 19], there is a lack of integrated understanding of adult experiences of AN treatments. The most recent metasynthesis of adult AN treatment experiences, drawn from studies between 1990 and 2005 [20], highlighted (a) a diverse range of participant treatment experiences; (b) that the exclusion of psychological dimensions of AN can limit the person’s engagement and investment in their treatment; (c) the complexity of psychological mechanisms in AN, particularly the concept of identity or who a person understands themselves to be and constructs a sense of self within a social context, that was argued to be one factor for the “lack of therapeutic success frequently reported in experimental studies and systematic meta-analyses” (p. 46); and (d) that more methodological rigor is needed. Furthermore, there is a need for more qualitative research that is interpretative (rather than descriptive) with more detailed analysis of the process (rather than only the outcome) of AN treatments.

Thus, the aim of this metasynthesis was to extend the previous reviews [20] to further understand key aspects of adult AN treatments, including facilitators and barriers to treatment and ways that questions of identity and personal agency are negotiated in treatments, to inform innovative AN treatments that generate improved outcomes that are also acceptable and preferable to the experiencing person. The focus of this metasynthesis was informed by the potential for chronicity in AN, the changing treatment needs over time [21, 22] and the findings of a previous metasynthesis that has proposed the absence of focus on the concept of identity as one factor in the lack of therapeutic success in AN treatments [20]. A specific objective of this metasynthesis was to understand ways in which adults negotiate a durable sense of identity and personal agency, including the identity positions [23] available to them to choose and live their preferred identity/ies, within treatment contexts.

## Methods and materials

The study was registered with Prospero (ID: CRD42018089259) and is reported according to PRISMA guidelines [24] https://www.crd.york.ac.uk/prospero/display_record.php?RecordID=89259.

### Identification and selection of studies

Research papers were included if they met the following criteria:

- Qualitative or as part of a mixed-methods design;
- Focused exclusively or partially on experiences of outpatient and inpatient AN treatment experiences;
- Involved the collection of original data with participants aged 18 years and over with a current or former diagnosis of AN.
  Research papers were excluded [18] if they were:
- Published in languages other than English;
- Not published in peer-review journal; and
- Did not include participants with AN.

Electronic databases searched included PsychINFO, MEDLINE, CINAHL, SCOPUS, and Web of Science, following the consultation with a health librarian. The dates searched were for all years until 17th February 2020. This search strategy included all years and diverged slightly from the pre-registration of this review with Prospero. Given the paucity of qualitative studies in this research area, the search strategy to include all years was designed to optimise the number of papers that met criteria for the review. A search strategy was used, combining the following key terms such as (anorexi* OR anore*) AND (intervention* OR treatment*) AND (qualitative*).

The title and abstract of each paper were individually evaluated by three reviewers (MP, JC, TM) for their relevance and adherence to the research criteria. A review of abstracts and a reading of full-text papers assessed for inclusion criteria by two reviewers (MP, TM) with three reviewers (MP, TM and CJ) screening the final full-text papers and any discrepancies in selection noted and resolved through discussion.

### Data extraction

Original qualitative transcript data in the form of all reported de-identified participant quotes (using pseudonyms chosen by original papers) that related to AN treatment and recovery experiences were extracted from each of the studies and coded for analysis with NVivo **©** [25]. No additional data were requested from the original investigators.

### Study quality assessment

The quality of papers included for review was assessed independently by three reviewers (MP, CJ, TM) and reported using criteria adapted from the Consolidated Criteria for Reporting Qualitative Research (COREQ; [26]) and a quality assessment tool for mixed methods (QATSDD; [27]) and any discrepancies in the ratings were resolved through discussion. No papers were excluded from this review based on their quality assessment score. The rationale for this decision was because all the studies reported rich qualitative data, regardless of their quality assessment score.

## Summary measures

The principal summary measure in the studies was in the form of themes and subthemes that were generated from methods including thematic analysis (6 studies), and interpretative/phenomenological analysis (5 studies) with a hermeneutic philosophy (1 study) or elements of grounded theory (2 studies) (Table 1).

**Table 1:** Characteristics of studies included in the metasynthesis

Source paper	Year	Country setting	Sample: size (N), age (years), %Female(F) /male (M)	Treatment type	Treatment focus	Recruitment	Data collection (OESSI = open-ended semi-structured interview)	Data analysis
Darcy et al. [50]	2010	USA	N = 24 19 – 52 years F = 100%	IP, OP	Unspecified	Community sources and online	OESSI and structured self-reported ED questionnaires	TA
Eli [37]	2014	Israel	N = 13 18 – 38 years F = 92%, M = 8%	IP	Unspecified	Community sources, online, treatment settings	OESSI—narrative: sensory experience and meaning making	IPA
Fox and Diab [46]	2015	UK	N = 6 19–50 years F = 100%	IP	> 2 psychological therapies	Two eating disorder services	OESSI	IPA
Gulliksen et al,. [42]	2012	Norway	N = 38 18–51 years F = 100%	IP, OP	Unspecified	Treatment settings	OESSI	GT
Gulliksen et al. [7]	2015	Norway	N = 34 18–51 years F = 100%	IP, OP	Unspecified	Treatment settings	OESSI	GT
Hannon et al. [45]	2017	UK	N = 5 23–30 years F = 100%	OP	AN Intensive Treatment Team model: intensive community-based care (> 2 years)	Treatment settings	Semi-structured interviews	IPA
Lose et al. [47]	2014	UK	N = 15 Age: 20 + years F: N/A	OP	MANTRA or SSCM	MOSAIC RCT site [34]	OESSI: on experiences of MOSAIC	TA
Rance et al. [38]	2017	UK	N = 12 18–50 years F = 100%	IP, OP	Cognitive behavioral therapy, cognitive analytic therapy, psychodynamic therapy and integrative therapy	Online, ED service	OESSI	TA
Ross and Green [39]	2011	UK	N = 2 Age: 18 + years F = 100%	IP	Psychodynamic inpatient treatment	ED service	OESSI	TA
Sly et al. [48]	2014	UK	N = 8 18–34 years F = 100%	IP	Treatment current	ED service	OESSI developed with themes from review of literature	IPA
Stockford et al. [41]	2018	UK	N = 6 33–48 years. F = 100%	OP	Unspecified	ED service	OESSI	IPA
Smith et al. [40]	2016	UK	N = 21 18–41 years F = 100%	IP	Treatment current	ED service	OESSI	TA
Wright and Hacking [49]	2012	UK	N = 6 21– 44 years F = 100%	IP, OP	Outpatient day program treatment current	ED service	OESSI	TA
Zainal et al. [43]	2016	UK	N = 148 Aged 20 + years F = 98%; M = 2%	OP	MANTRA [35] or SSCM [36]	ED service	Self-report questionnaires/feedback forms Collected 12 months post treatment	TA

*IP* inpatient, *OT* outpatient, *GT* grounded theory, *IPA* interpretative phenomenological analysis, *TA* thematic analysis

### Meta-synthesis

The final 14 full-text papers were each read multiple times by two reviewers (JC, CJ) to become familiar with the texts. A thematic synthesis [28] was conducted that drew on the qualitative method of modified analytic induction [29] (more recently renamed deductive qualitative analysis [30]). This involved extracting the qualitative data extracts provided by each selected study, line-by-line coding (independently by JC and CJ), engaging in a process of constant comparisons between these initial codes to generate four descriptive themes.

In the final stage of the synthesis, the descriptive themes were analysed to generate broader third order analytical themes [28] using the sensitised concept [29, 30] of identity, proposed by an earlier metasynthesis on adult AN treatment experiences [20], to anchor the analytic process to a prior substantive research finding [20]. These themes were discussed with the other authors (TM, PH) until a final version and thematic map were agreed upon. Although potentially the most controversial aspect, due to its dependence on the judgment and insight of the reviewers, this final step of going beyond the content of the original studies is widely considered to be the defining characteristic of a thematic synthesis [31, 32]. The emphasis of this stage of the meta-synthesis included an interpretive review that was inevitably shaped by the researchers’ positioning, subjectivities and values that also allowed for a critique of current thinking to be questioned on how adults experience AN treatments [33]. Data within these themes were then recursively analyzed to broadly address the research aims related to barriers/facilitators to engagement with therapeutic services, and identity and personal agency negotiation within the context of AN treatments and processes of recovery.

## Results

### Study selection

**Fig. 1:**
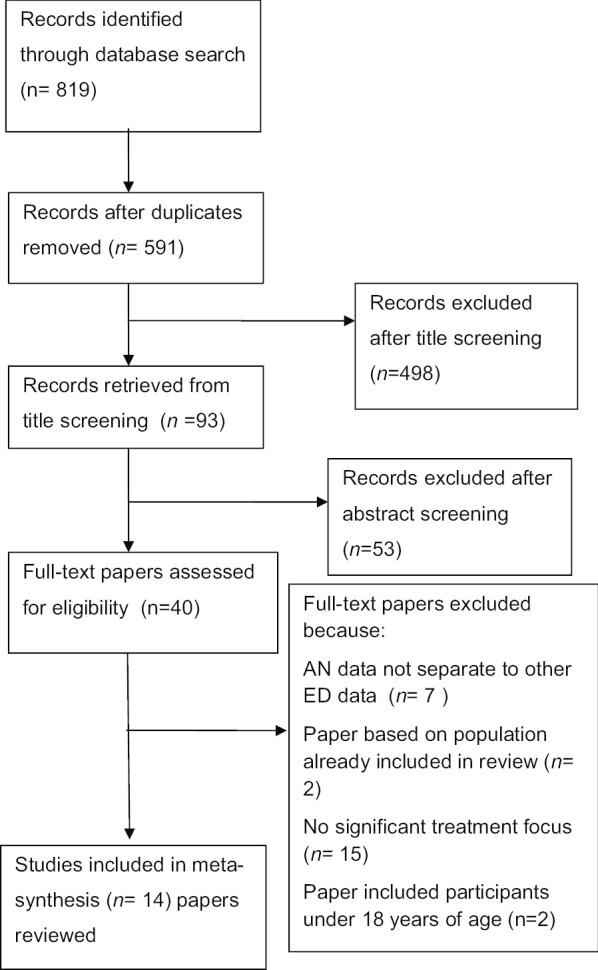
Flow chart of search strategy. Database search strategy for the identification of research papers that met criteria for inclusion in the present metasynthesis

The initial screening by title resulted in 88 papers. A review of these abstracts narrowed the selection to 37, while a reading of the full-texts yielded 14 papers that met inclusion criteria (Fig. 1). The identified papers were all published between 2010 and 2018.

### Study characteristics

Table 1 outlines the characteristics of the 14 selected studies.

### Quality assessment of selected studies

Fourteen studies met the search criterion. Quality assessment of the studies (Additional file 1: Table A) found that the most frequent reason for a low score was lack of pilot data of interview (9 studies); followed by only one coder employed (7 studies) and no member (or participant) checking of data extraction/analyses (6 studies).

### Risk of bias across and within studies

Potential biases across studies included: inconsistency in the time point of the data collections (during, immediately post treatment, and retrospectively); lack of consideration for key factors such as inpatient and outpatient context, illness severity, and a number of therapies; lack of inclusion of included interview questions to assess for potentially biasing the data and the interviews’ direction; the selection of illustrations that provided only a partial access to the studies’ data; an overreliance on single coders/data analysts and under-reliance on participant member checking (see Additional file 1: Table A); and the absence of discourse analysis methodologies across the studies. Within the studies, there was an absence of male participants, gender and cultural diversity. Treatment context may also have influenced participant experiences for example, inpatient treatments may be more prohibitive and focus more on eating behaviour change/symptom management to mitigate medical risk.

### Results of individual studies

The 14 studies generated diverse themes ranging from participant choice and collaboration, ambivalence and adjustment to change, the meanings of ascribed to AN and recovery, preferences for a working therapeutic alliance, and responses to the focus of various treatment interventions (Additional file 1: Table B). The majority of these themes were descriptive with five of the studies generating themes that went beyond the data and related more specifically to questions of identity [37–41].

### Synthesis of results

**Fig. 2:**
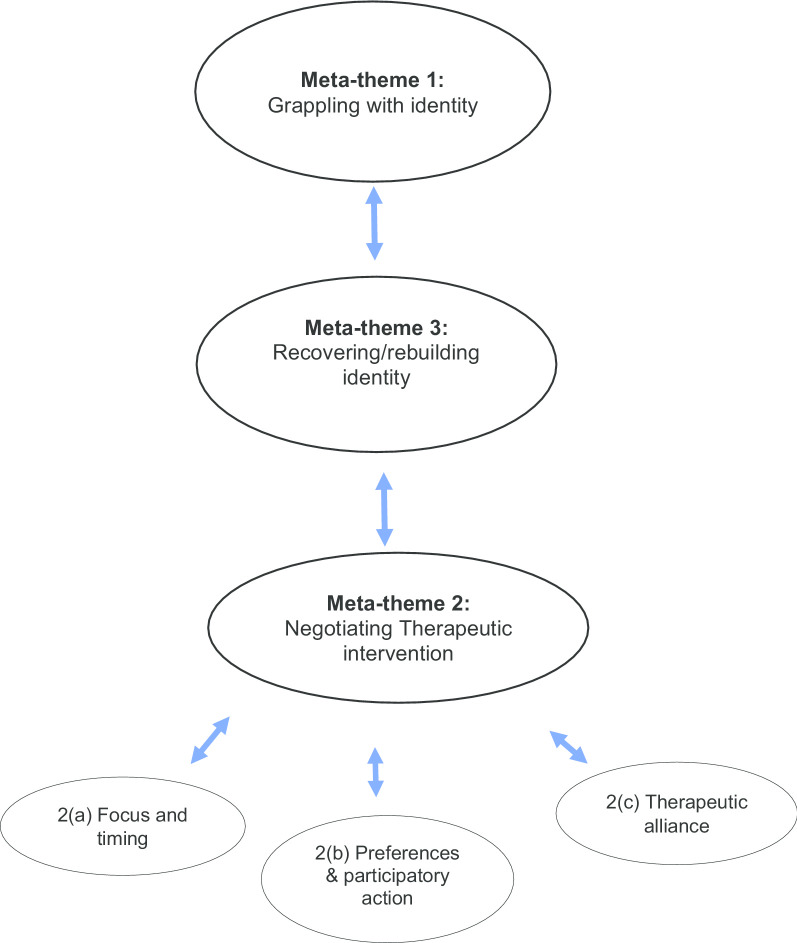
Relationship between meta-themes and subthemes of AN treatment experiences. Schematic that illustrates the inter-relationship between AN treatment experiences and the sensitised concept of identity

Based on the qualitative data extracted from the 14 studies, a series of interconnected meta-themes were identified, and their inter-relationship are depicted in Fig. 2.

#### Meta-theme 1: grappling with Identity

Participants ascribed diverse meanings to the AN experience and in doing so, they actively engaged in patterns of identity negotiation (See Additional file 2: Table C for exemplar quotes). Within these contexts, participants engaged in discernment of whether AN was problematic (or not) and/or part of my identity (or just me). Significant in these identity negotiations was the extent by which personal agency was experienced by participants to negotiate and ultimately choose their preferred identity/ies.

Across all the studies, the participants experienced ambivalence in relation the question of whether AN was problematic, particularly when speaking about the egosyntonic effects of AN, including a sense of pride, achievement and control that was hinged upon the thin body. On the other hand, AN was experienced by some participants as related to a lack of self- worth, for example, the sense that “you don’t think that you deserve anything” (Beth) [41, p. 132], a solution to dealing with distress, yet also leading to distress because of the negative impact of AN on the person and their relationships [41].

Being totalized with the identity of a sick person who had anorexia was a frequent dilemma [42]. This location of their experience as a disease or illness was, however, preferred by some participants over conceptualisations such as “something wrong with me as a person” (P16) [43, p. 6]. On the other hand, diagnosis of an eating disorder, particularly when experienced as a textbook approach [38], contributed to a loss of identity to the anorexic identity for some participants across four of the studies [37, 38, 40, 42]. This struggle to locate the AN experience as problematic was not confined to the individual but also inadvertently and systemically strengthened through (1) recollections of parents struggling to discern their child’s struggles as AN [41]; and (2) accounts of not being taken seriously by treatment systems contributing to the sense that they were not thin or ill enough to qualify for treatment [42].

Furthermore, the question of personal agency in the AN experience (e.g. “chosen myself”; Alicia [7, p. 211]) impacted on whether or not the person perceived their experience to be an illness or not [44].

*EXTRACT 1:*

*Vered: I’m not worthy of this title, I didn’t spend enough on it… not enough blood, not enough tears, not enough suffering* [37, p. 4].

Extract 1 exemplifies the moral discourse that constructed the identity of anorexic where qualifying for “this title” was also contingent on the extent of personal investment and suffering. As participants negotiated the question of whether they qualified as sick enough, had chosen and/or were to blame (Denise) [45] for AN, they were also faced with the question whether AN was part of their identity or not.

AN was experienced as both an identity investment and a troubling entrapment across the studies. In ten of the 14 studies, participant narratives highlighted the dilemma of whether AN was separate (e.g. as an eating disorder/illness) and/or part of participants’ identities [7, 37, 39–41, 43, 46–49]. The struggle to give up AN included the question of how to define oneself, which included giving up the identity as a good anorexic (Carla) [46], the control [40, 46], power [48], safety and a distraction to cope [41] that was generated through the experience.

*EXTRACT 2:*

*Helen: When I was so ill I felt like I was two people. I’d got the anorexia and I’d got me, and I was really confused, and it was a battle […] I know who Helen is but where does the anorexia and the negativity fit? I know it’s there but where does it fit in to make the whole person?* [39, pp. 114–115].

This internal struggle, exemplified in extract 2, generated a fragmented sense of self with an internal battle between different parts of herself, yet also a desire to experience and see herself differently. Within this text, Helen was engaged in a process of identity negotiation, where she was faced with a number of identity positions from which to choose her preferred identity/ies, including the implicit question of who am I if AN is no longer me? A therapist’s stance was significant in these contexts, including the extent by which they stood with the person in these identity negotiations.

#### Meta-theme 2: negotiating therapeutic intervention

Meta-theme 2 focuses on key components that participants negotiated within therapeutic contexts, including treatment focus and timing, personal preferences, participatory action and the therapeutic relationship (see Additional file 2: Table D for exemplar quotes).

#### Subtheme 2(a): therapeutic focus and the significance of timing

The question of preferred therapeutic focus was diverse across the 14 studies, particularly the question of whether this was best placed on addressing symptoms and/or the onset, context and meaning of the AN experience. Participants raised the question of therapeutic structure and the timing of highly structured ED treatments (particularly inpatient contexts) that, although potentially life-saving and helpful, also resulted in diminished personal agency and, for some, were experienced as traumatic [37, 41]. Furthermore, the absence of treatment focus on what was important to the person was potentially highly detrimental and the importance of a therapist’s skill in discernment of the timing of these interventions within the context of an overall treatment plan.

*EXTRACT 3a:*

*Megan: [I] spent two years with them [local ED service] … to get to the point where I realized that focusing on food and maintaining restoring weight doesn’t work for me, because all it does is push me further into depression … which I don’t handle very well … it makes me suicidal* [38, pp. 587–588].

*EXTRACT 3b:*

*Joanna: You feel very alone, you put weight on and then you’re told you can go when you’re struggling the most with your weight. Then you’re on your own, scared, afraid of being a woman, afraid of assumptions being made by others that you’re OK* [39 p. 114].

Extract 3a highlights a significant risk of a treatment focus on symptom reduction without attending to the emotional holding of the person. Furthermore, when the physical crisis and treatment support was over, the psychological crisis re-emerged. Extract 3b highlights how this psychological crisis included an ongoing grappling with questions of identity in this context of diminishing therapeutic support. A number of participants across the studies resisted structured and behaviorally focused treatments because of the frequently disputed assumption that recovery was achieved at the point of weight restoration and eating behavior change.

#### Subtheme 2(b): personal preferences and participatory action

The significance of tailoring treatment interventions to a person’s self-identified needs and preferences was highlighted in the participant narratives in 10 of the 14 studies [7, 37, 39–43, 45, 47, 48]. Implicit within the tailoring of therapeutic interventions was the power dynamic between the therapist and the person and the extent by which the person experienced themselves as an active participant who had a voice within the therapeutic dialogue.

*EXTRACT 4:*

*Johanna: It’s good that someone challenges you to do things that you might not always feel capable of handling. And then to be there for me…also when things get really hard* [42], p. 937].

This extract highlights how implicit in a therapist’s challenge was the belief that the person has the capacity to change. When participants recounted being active participants in treatment this was perceived as their contributions were understood as valuable; treatments were more effective and empowering.

On the other hand, in arguing for the significance of tailored approaches to their treatment, participants across these 10 studies highlighted the detrimental effects of treatments that were rigid, not linked to their lived experience (exemplified in extract 5a) and implicitly blamed them (exemplified in extract 5b) if improvements did not eventuate.

*EXTRACT 5a:*

*Diane: […] When that [treatment] didn’t work out, I thought; there is no hope for you.[…] I wasn’t able to understand the connection between what he was talking about and my everyday life* [7, p. 216].

*EXTRACT 5b:*

*Claire: It felt quite rigid […] and if you’re not getting better you’re just not trying hard enough’* [38, p. 588].

The urgency to address the physical crisis of AN posed a risk to the basic therapeutic alliance for a number of participants across the studies.

#### Subtheme 2(c): therapeutic alliance: journeying together and being treated as a person

The question of therapists’ capacities to journey with the person was evident in the narratives across the studies. Rather than an individual pursuit, change was experienced within the interpersonal context of the therapeutic relationship that supported readiness and stability. The detrimental effects of an absence of a therapist holding and understanding was also present in some participant narratives across all the studies: for example, “the punitive thing … fuels the disease” [50, p. 267]. On the other hand, present in three of the studies [7, 37, 49] was the impact of the dialogical space of therapy that enabled some participants to expand their vision of themselves: depicted by one participant as “they give me eyes” [49, p. 112]

Narratives across the 14 studies indicated that participants were active in discerning whether the therapist was someone they could trust. The most accessible way that trust was understood was in the person of the therapist and their expertise. Contrastingly, across half of the studies [7, 37, 38, 40–42, 46], participants highlighted the significance of therapists’ trust in them and being treated as a person, exemplifying that trust is implicitly a two-way process.

*EXTRACTS 6:*

*Ava: […] this therapist is that he treats me like someone, even though he sees that I have a disturbance, he treats me like a person who is able to achieve things in life, who can manage to get rid of this* [42, p. 937].

*Emma: […] to have people that care for you and accept you and that don't look down their nose at you and ya know see you as a worthy human being that's an individual and that can spend time with you not just go shovel you full of cheese and fattening foods with no humanity* [41, pp. 135–136].

Being treated as a person who had a range of self-capacities, recursively cultivated trust in the therapist, and contributed to the sense that there was more to the person than the ED, that their struggles were taken seriously and that they were worthy of care. This parallel process of a therapist’s trust in the person’s capacity to change was also evident when there was a perceived absence of this, that had profound effects, for example with some participants reportedly giving up on themselves at that time [46].

Significant in the participant narratives was the capacity of therapists and treatment teams to cultivate a therapeutic relationship built on understanding and regular checking in with the person to prioritise their voice on matters related to the ED experience and its treatment.

#### Meta-theme 3: recovering and rebuilding Identity

Participant narratives highlighted the diverse ways that they engaged in rebuilding a sense of identity through insights into themselves and recovering a sense of identity from the anorexic identity (see Additional file 2: Table E for exemplar quotes). Insights into AN were highlighted by participants in four of the 14 studies as significant in their recovery [39, 43, 45, 47].

*EXTRACT 7*

*P32: I feel that the therapy has given me a much greater understanding of why I keep this illness and what the illness gives me. I strongly believe that I needed this insight to be able to work on recovery* [43, p. 6].

Insights included the conceptualization of the AN experience as a journey of (1) self-understanding; (2) facing and voicing emotions (e.g. “instead of letting the eating disorder do the talking” (P1) [43, p. 6]; (3) understanding emotions as a response to life and history (e.g. “it really did help me find out who I was, all the anger coming out, all the negativity. It was the first time I felt safe expressing that” (Helen) [39, p. 116]*;* and (4) reconstruction of meaning around painful life experiences.

Participants took up diverse positions on recovery from completely recovered [43] to a lifelong struggle (“for the rest of my life” (Hayley) [38, p. 589]). In half of the studies [38, 40–43, 46, 50], participants found the dualistic construction of recovery, as either the presence or absence of disorder, to be troubled. Ongoing struggles contributed to the sense that recovery was difficult, if not unobtainable, even when the person desired the ED to be gone. The stance of treatment teams was important in these negotiations as highlighted in the following extracts:

*EXTRACTS 8:*

*Ava: And he [therapist] doesn’t have the perspective that first we have to get rid of this eating disorder and then you can start your life. It’s more the perspective that it’s possible living with this disturbance and doing things along the way, you know. He puts a lot of faith in that you can get out of a disturbance by starting to live your life differently* [42, p. 937].

*Helena: I want them to give them the same treatment opportunities as people they’ve see for the first time, so to keep believing that recovery is possible for anyone* [41, p. 136].

These participants argued for the importance of therapists being able to stand with them in hope to reclaim their lives from AN and its effects, regardless of it duration and severity.

This focus on living life differently and in doing so rebuilding connecting a sense of oneself as more than the AN was consistent with participant narratives in five other studies [39, 40, 43, 45, 47].

*EXTRACT 9*

*Participant 15: I am getting character in myself again and finding a personality and it is all coming back to me who I want to be* [40, p. 23].

This extract depicts how some of the participants experienced recovery as a process of reclaiming lost identities through re-engaging in life in valued ways.

## Summary: inter-relationship between meta-themes

The therapeutic relationship provided an interpersonal context within which participants engaged and negotiated processes of meaning making of their lived experience and rebuilt and recovered a sense of identity from AN and the treatment interventions that facilitated this.

## Discussion

This present metasynthesis extends the findings of previous metasynthesis [20], including some of the ways participants’ negotiated (1) meanings of AN experience; (2) their identities within AN treatment contexts; and (3) rebuilding their identities. Moving beyond a descriptive synthesis, this present paper has sought to understand how participants were active in their responses to AN and its treatment and implications for both when and how treatment is delivered within a treatment non-negotiable context that prioritises safety.

This metasynthesis supports the view from findings in adolescents [18], that change in AN co-exists with significant ambivalence and the enabling of personal autonomy and agency in the type and timing of treatment interventions was important to adult participants across the studies in this metasynthesis. The question of when and how to invoke structured interventions to address AN symptoms was argued differently by participants both within and between the studies; most concerningly was when interventions were experienced as traumatizing or contributing to a loss of hope for the person with a lived AN experience. Furthermore, participant treatment experiences highlighted the importance of the focus of treatments shifting from modality alone to the dynamic intersection between the treatment type and the therapeutic relationship. This is consistent with Espindola and Blay’s [20] metasynthesis that found that one factor that facilitated change was “satisfactory affective relationships”, which did not merely act as support but rather as “active interventions” (p. 45).

### Summary of evidence

The evidence has been summarized in the form of therapeutic challenges and key clinical applications for adult AN treatments:

*Key therapeutic challenges*

- Addressing individual differences and balancing treatment interventions to prioritise physical safety and assist the person to find their own identity, including a sense of self-worth and that they are more than the AN;
- Preserving personal agency and autonomy within a treatment non-negotiable context and the timing of interventions.

*Clinical applications and considerations*

- Checking in with the person and being responsive to their therapeutic needs and preferences within a treatment non-negotiable context that prioritises safety;
- Establishing a respectful therapeutic alliance that includes being held emotionally, treated as a person who has a range of self-capacities and is more than the AN, and instils hope; and
- Journeying alongside the person in their recovery, including engaging in what recovery means to the person and addressing processes of rebuilding identity.

#### Therapeutic challenges

Across these studies, a number of therapeutic challenges remain in the treatment of AN.

##### Addressing individual differences and questions of identity

There was little consensus amongst participants about what type of treatment intervention was most helpful. The delicate balance between interventions that focus on physical safety (eating behavior and weight restoration) and those that address psychological distress was evident across the studies. Interventions that primarily focused on physical safety posed a risk to the basic therapeutic alliance, particularly if perceived as punitive, traumatic and as reproducing a sense of despair (including suicidality for some) associated with the AN experience itself [7, 37, 38, 50]. Further problematic was when there was a misalignment between what a successful treatment looked like for person delivering treatment and the person receiving treatment. On the other hand, treatments that both prioritized physical safety and assisted a person to rebuild their own identity with the recognition that they were more than the AN were positively perceived and generated identity conclusions that included a sense of self-worth. The question of how to address individual differences would also benefit from more moderator and mediator analyses to determine what worked best for whom and when.

##### Personal agency in treatment negotiation

When and how to tailor treatment to the person, particularly in contexts where urgent life-saving nutritional restoration is required and/or the person is unwilling or unable to change, continues to pose a dilemma for clinicians. Geller and Srikameswaran [16] have argued for due consideration being given to the delivery of treatment non-negotiables to optimise treatment experiences and outcomes. Significant to participants across the studies in this metasynthesis was the therapist stance of journeying with them in ways that were experienced as empowering, and cultivated a sense of personal agency, including discursive agency [44] in the construction of meaning in the AN experience and its recovery. Overall, the question was less about the type of intervention but rather, the timing of particular interventions and how (and whether) this was negotiated with the individual person [7, 37, 38, 41, 47].

### Clinical applications and considerations

Key applications of this metasynthesis that has focused on the perspectives of people with a lived AN experience include the importance of personal agency that was experienced within the context of tailored treatments within a non-negotiable framework, where implicit in being challenged to change was a sense of hope that change was possible, and therapists journeying alongside the person as they recover a sense of identity outside the anorexic identity.

- *Checking in and tailored treatments:* The responsiveness of therapists and treatment teams to both the needs and preferences of the person within a treatment non-negotiable context is key in the experience of personal agency in treatment. Future AN therapeutic interventions and research need to consider how client feedback, through regular checking in, may not only build in flexibility to tailor interventions but also cultivate the sense that the person’s views (and they) matter. Routine collection of client feedback has been found to be an essential component of any evidence-based therapeutic practice; for example, feedback informed therapy has been found to double treatment effect sizes, reduce in treatment drop-out rates and shorten the length of treatment interventions [51].
- *Therapeutic alliance*: The therapeutic relationship is becoming increasingly recognised as a significant dimension of treatment effectiveness, at least as, if not more, significant than the therapeutic modality [52] with the crafting of these being the essential element of therapeutic effectiveness. Key aspects of the therapeutic relationship that were synthesised across these qualitative studies centred on a therapist: being with the person throughout their journey in a way that they felt held emotionally without being overwhelmed by AN; trust and hope for the person in recovery; and challenging the person to see that they were more than AN and to engage them in the therapeutic work of questioning who am I as a person if AN is no longer dominating my life [53]. Being treated like a person who had a range of self-capacities also recursively cultivated a sense of trust in the therapist and contributed to the sense for the participants that there was more to them than the AN, that their struggles were taken seriously and that they were worthy of care.
- *Recovering identity:* Notable for participants across the studies was whether therapists joined (or clashed) with their processes of meaning making and journeys of rebuilding their lives and identities from AN and its effects. The studies in this metasynthesis highlighted “hope and empowerment” [54, p. 1010], that was aligned to the recovery model, including “the development of new meaning and purpose in one’s life as one grows beyond the catastrophic effects of mental illness” [55, p. 11]. Implications for therapists include discernment of what a minimum recovery level is for a person to experience a livable recovery. This is where the shift moves from eradication of symptoms for all towards quality of life and re-instatement of a sense of meaning, purpose and finding their own identity.

In summary, the provision of respectful therapy that enables the person to disclose their ED experience [56], including in their own experience-near terms [44, 57]; instils a sense of hope for recovery [58]; and supports their recovering and rebuilding of identity outside the AN identity [57, 59] need to be primary considerations in the development of AN treatments. Given the significant rates of drop-out and distress in people undergoing treatments for AN, emotional holding, therapeutic alliance and shared commitment, regardless of the therapeutic modality, were experienced by participants across the studies as essential dimensions of the ED treatment experience and its effectiveness.

### Limitations and strengths

Through the process of synthesising the data from the included papers, researcher judgements of credibility and contribution of the papers were made. The absence of treatment experiences reported by participants in research studies: (1) in languages other than English; and (2) in grey literature, including in the form of unpublished data; places limitations on the scope of this metasynthesis. Furthermore, there was a significant amount of transcript data not reported in the published papers that were unavailable to be synthesized in this study and there are thus likely to be gaps in treatment experiences that are not captured in this metasynthesis. Furthermore, the themes generated in this paper are influenced and shaped by the focus of this metasynthesis on the sensitized concept of identity.

Despite these limitations, this paper has a number of strengths including being a response to the paucity of updated research in the lived experience of AN treatments and in the context of AN treatments having inconclusive outcomes as to what works in treatment. Furthermore, this metasynthesis included several of the authors working on steps in parallel in analysis of data drawn from a number of studies. By drawing on the voice of those with a lived AN experience across varied adult treatment contexts, the findings of this paper generate a map for therapists as they balance their treatment interventions between nutritional and physical safety and assisting the person to find their own identity.

### Future directions

There is a need to research AN interventions that have scope to be flexibly tailored to the needs and preference of the person with a lived experience and to develop a richer understanding of the complex interaction between therapeutic alliance and treatment interventions. Furthermore, future studies would benefit from including more diverse participants, especially men and people of diverse gender, sexual orientation and cultural backgrounds, a broader focus beyond the dichotomies of helpful and unhelpful experiences, a greater triangulation of findings and addressing timing in addition to treatment modality. Qualitative research in this area needs to move more comprehensively beyond description towards in-depth data analysis, with the understanding of participants as active as they speak [60] and in their identity negotiation [61] within the dialogical space of treatments and research.

### Conclusions

This review concludes that whilst one-sized treatment may not fit all in AN, when it comes to the therapeutic relationship, participants consistently preferred being treated as a person (who was more than the AN) who had skills and capacities in being able to contribute to what was needed and when. At times during their treatment, participants appreciated therapist expertise and challenge where implicit in this, was hope in their capacities to recover their lives from AN. Also pertinent for participants was therapists who demonstrated genuine interest and concern in them as a person beyond the AN experience and that this had profound impacts on expanding their vision of themselves.

Future research is needed into the complex interpersonal dynamics of the therapeutic relationship, including the question of what works and when for the person with a lived AN experience. There needs to be scope for therapists in both research and therapeutic contexts to draw on their clinical expertise to individualize treatment interventions to the experiencing person, particularly as this has been found to act as a potent contributor to psychotherapy and treatment outcomes [52]. Future research into the development of AN treatments and outcomes would also benefit from increased participatory-action research that privileges the voice and perspectives of the person in matters related to their own treatment [55] and prioritises both alternative ways of being and the rebuilding of a sustainable sense of identity in the context of the complex social reality of the AN experience.

## Supplementary information

**Additional file 1: Table A**. Assessment of quality of included papers; **Table B**. Summary of themes and findings identified in included studies.

**Additional file 2: Tables C, D & E**. Exemplar data extracts for Metathemes 1-3 in the Metasynthesis.

## Data Availability

The additional files document contains the following data and materials for the metasynthesis: Assessment of quality of included papers (Additional file 1: Table A); Summary of themes and findings identified in included studies (Additional file 1: Table B); and Exemplar data extracts for Metathemes in the Metasynthesis (Additional file 2: Tables C, D and E).

## Abbreviations

AN: Anorexia nervosa
COREQ: Consolidated Criteria for Reporting Qualitative Research
ED: Eating disorder
GT: Grounded theory
IP: Inpatient
IPA: Interpretative phenomenological analysis
OT: Outpatient
PRISMA: Preferred Reporting Items for Systematic Reviews and Meta-Analyses
QATSDD: Quality assessment tool for mixed methods
SSCM: Specialist Supportive Clinical Management
TA: Thematic analysis
