# Supplementary material for: “Finding my own identity”: a qualitative metasynthesis of adult anorexia nervosa treatment experiences
Source: BMC Psychol. 2020 Oct 22;8:110. doi: 10.1186/s40359-020-00476-4 (PMC7583290; doi:10.1186/s40359-020-00476-4)
Supplement: Supplementary file 1 — Additional file 1: Table A. Assessment of quality of included papers; Table B. Summary of themes and findings identified in included studies. [file 40359_2020_476_MOESM1_ESM.docx]

## ADDITIONAL FILES

Table A: Quality Assessment of Papers included in Metasynthesis

| **Author(s) (year)** | Darcy (2010) | Eli (2014) | Fox & Diab (2015) | Gulliksen, et al. (2012) | Gulliksen et al. (2015) | Hannon, et al (2017) | Lose, et al. (2014) | Rance, et al (2017) | Ross & Green (2011) | Sly, et al. (2014) | Smith, et al. (2016) | Stockford et. al. (2018) | Wright & Hacking (2012) | Zainal, et al. (2016) |
| --- | --- | --- | --- | --- | --- | --- | --- | --- | --- | --- | --- | --- | --- | --- |
| 1. **Clear theoretical framework** | 2 | 3 | 0 | 2 | 2 | 1.5 | 1 | 1 | 1 | 1 | 1 | 3 | 2 | 1 |
| 1. **Data collection tool appropriate to aims** | 3 | 3 | 1 | 3 | 3 | 1.5 | 3 | 3 | 2 | 2 | 2 | 2 | 2 | 1 |
| 1. **Piloting of data collection tool.** | 2 | 0 | 0 | 2.5 | 3 | 0 | 0 | 0 | 0 | 0 | 2 | 0 | 0 | 0 |
| 1. **Representative sample size.** | 3 | 3 | 1 | 3 | 3 | 2.5 | 3 | 2 | 0 | 2 | 3 | 2 | 1 | 3 |
| 1. **Method of approach outlined** | 2 | 3 | 1 | 3 | 3 | 2 | 3 | 2 | 3 | 2 | 3 | 3 | 2 | 2 |
| 1. **Sample adequately described** | 3 | 1 | 2 | 3 | 3 | 3 | 3 | 1 | 2 | 1 | 3 | 3 | 1 | 3 |
| 1. **Clear and appropriate method of data analysis** | 3 | 2 | 1 | 3 | 3 | 2 | 2 | 1 | 1 | 3 | 3 | 3 | 2 | 2 |
| 1. **Data triangulation** | 3 | 2 | 0.5 | 2 | 0 | 1 | 3 | 1 | 2 | 3 | 2 | 2 | 1 | 3 |
| 1. **Member checking** | 1 | 1 | 2.5 | 0 | 0 | 0 | 2 | 2 | 0 | 3 | 2 | 0 | 0 | 2 |
| 1. **>1 data coders** | 1 | 0 | 0 | 0 | 0 | 3 | 2 | 1 | 0 | 2 | 0 | 2 | 0 | 3 |
| 1. **Data synthesized** | 3 | 2 | 2 | 2 | 2 | 2 | 3 | 1 | 2 | 2 | 2 | 3 | 3 | 3 |
| 1. **Sufficient original data provided** | 3 | 1 | 1.5 | 2 | 2 | 2 | 2 | 1 | 2 | 2 | 2 | 3 | 2 | 1 |
| 1. **Data and findings consistent** | 3 | 2 | 1 | 2 | 3 | 2 | 3 | 2 | 2 | 2 | 2 | 3 | 2 | 2 |
| 1. **Total Quality Assessment Score** | **H** | **M** | **L** | **M** | **M** | **M** | **H** | **L** | **L** | **M** | **M** | **H** | **L** | **M** |

0=none, 1=very slightly, 2=moderately, 3=complete

Total Quality Assessment Score: Low (L): < 20 ; Medium (M): 20-29; High (H): > 30

Table B: Summary of Themes and Findings Identified by Included Studies

| **Source Paper** | **Research Focus** | **Themes** | **Sub-Themes** | **Conclusions** |
| --- | --- | --- | --- | --- |
| Darcy et al., (2010) | How do individuals with AN engage in treatment and define recovery? | Relationship between choice and motivation.  Treatment goals.  Definition of Recovery.  Factors influencing dropout. | Treatment Choices.  External.  Against own will.  Referrals/doc’s recommendations.  Health insurance.  Family/Loved Ones.  Parents.  Joint decision with parents.  Other loves ones.  Self  Former.  Parents.  Personal.  Acknowledgement/to be heard.  Other.  Current.  Psychological/thoughts.  Social.  Nothing/DK.  Self-acceptance.  Family work.  Other.  Symptom Specific.  Eating comfortably.  No distorted thinking.  Normal physiological functioning.  General healthy functioning.  Body image/self-esteem.  No such thing.  Ambivalent.  Don’t know.  Recovery as ongoing.  AN easier than recovery.  Social/Interpersonal.  Social Relationships.  Confidence/Assertive.  Other misc. factors.  Setting Specific.  Specific therapists.  Punishment/ reward systems.  Inappropriate settings.  Other/setting specific.  Personal Factors.  Tired of it/too much.  Homesick.  Fear of weight gain.  Other personal. | Those with more involvement in treatment choice had better motivation to change and normalized eating. Participants’ definition of recovery mapped well to current research conceptualizations, though a substantial proportion of the group expressed some ambivalence around the concept. Results are interpreted in the context of self-determination theory of motivation. This paper suggests that patients should be involved collaboratively in the formulation of shared goals and concepts of recovery in treatment settings. |
| Eli (2014) | What are the subjective experiences of eating disorders and recovery among Israeli adults? | A sense of recognition and legitimacy.  Dynamics within the inpatient community.  Boundaries vis-a`-vis the ‘outside’ world and one’s own illness. | The eating disordered self.  The patient as an individual.  Seeing the self in others.  Dangerous others.  Sheltering boundaries.  Oppressive boundaries. | The study suggests that this ambivalent positioning can usefully be understood through the anthropological concept of liminality: being both a part of and apart from one’s community. |
| Fox and Diab (2015) | Exploration of the perceived experiences of living with and being treated within an Eating Disorders Unit for chronic AN (cAN). | Making sense of AN.  Experience of treatment.  Interpersonal relationships.  Battling with anorexia.  Staff pessimism in the treatment of chronic AN. | General relationships.  Relationships with staff/therapists | The paper highlights how the self is entwined with anorexia nervosa and thus making it incredibly difficult to perceive a life without cAN. |
| Gulliksen et al,. (2012) | Health professional characteristics preferred by AN-patients. | Acceptance.  Vitality.  Challenge.  Expertise. | Negative experiences with health professionals.  Disregard.  Prejudice.  Positive experiences with health professionals.  Generosity.  Respect.  Patience.  Negative experiences with health professionals.  Passivity.  Positive experiences with health professionals.  Active interest.  Sense of humour.  Negative experiences with health professionals.  Pampering.  Positive experiences with health professionals.  Focus on resources.  Support through difficult situations.  Positive experiences with health professionals.  Experienced knowledge.  Authoritativeness. | The study suggests that treatment of AN requires therapists who are capable of using a complex set of behaviours when interacting with their patients. |
| Gulliksen et al,. (2015) | Circumstances, reflections, and reactions to first treatment contact in women with AN. | Circumstances.  Reflections.  Reactions. | Initiative to conversation.  Family and friends initiated.  Self-initiated.  Health-professional initiated.  Challenges to treatment alliance.  Ambivalence to treatment.  Resistance towards recovery.  Help-seeking motives.  Wish to become a better anorectic.  Wish to feel less depressed.  Wish to reduce somatic concerns.  Loss of autonomy.  Feeling angry.  Feeling trapped  Communication skills.  Feeling not taken seriously.  Feeling taken seriously.  Feeling safe.  Knowledge of eating disorders.  Feeling disappointed.  Feeling less alone. | The results suggest that (a) health care professionals need to demonstrate effective professional communication skills and proficient knowledge about EDs in early contacts; (b) treatment goals other than recovery from AN should be explored; and (c) patients’ view of AN as a strategy for increased control and mastery in life may be a topic for discussion in the early contacts. |
| Hannon et al., (2017) | How do people experience long term community treatment? | Treatment experience  Function of anorexia  Self-criticism versus self-acceptance  Isolation versus connection  Hopelessness versus hope  Stuckness versus change |  | Full recovery from anorexia is possible with intensive community therapy. Patients with severe and enduring AN expressed a positive experience of a unique community therapy. |
| Lose et al., (2014) | Evaluate patients’ treatment experiences of MANTRA and SSCM. | MANTRA.  SSCM.  Shared factors. | Positive and helpful aspects.  Structured and flexible approach.  Helpful manual.  Other helpful aspects.  Beneficial outcomes.  Altered stance to feelings and thought processes.  Improved communication and confidence.  Improved quality of life.  Less helpful aspects.  Issues with the manual and treatment.  Possible improvements.  Changes to treatment, manual and frequency of sessions.  Practical advice and prescriptiveness.  Positive and helpful aspects.  Practical and proactive approach.  Focus on nutrition.  Beneficial outcomes.  Learning to move forward.  Understanding eating disorder symptoms and their impact on behaviour.  Improved communication and confidence.  Improved quality of life.  Less helpful aspects.  Focus on weight and eating.  Limited therapeutic content, continuity and structure.  Possible improvements.  Changes in therapeutic content, process and structure.  Treatment duration and intensity.  Positive and helpful aspects.  Regularity and predictability.  Talking therapy.  Less helpful aspects.  Delays and disruptions.  Therapeutic and external environment.  Therapist to patient match.  Therapist’s characteristics and approach.  External circumstances | The findings show clear differences and some overlaps between patients’ views on MANTRA and SSCM. Both therapies were experienced by patients as credible and largely helpful, albeit in different ways. These results are in agreement with those of therapists’ views on these treatments. |
| Rance et al., (2017) | Eliciting the treatment experiences of people with AN. | Access to treatment  Treatment structure  Treatment providers  Being treated as a ‘whole person’ and having a ‘real’ relationship |  | Women were dissatisfied with treatment and perceived it to be focused and driven by food and weight. Women had a negative view of CBT as too rigid and focussed on weight.  What the women wanted was to be treated as a ‘whole’ person and have a real relationship with their therapist. Women’s experience of inpatient treatment was it lacked psychological therapy and outpatient therapy was too short. |
| Ross and Green (2011) | Can inpatient admission for AN be therapeutic? | Relationship to their illness.  Relationship to the unit.  Relationship to themselves.  Relationship to others. |  | This study indicates the use of therapeutic approaches that emphasise the importance of the therapeutic relationship in the treatment of chronic anorexia nervosa. It is suggested that psychodynamic and attachment theories should be used to inform overall treatment models. |
| Sly et al., (2014) | Examination of the experiences of service users in developing therapeutic alliance whilst in treatment for AN. | Alliance as a key experience.  Being active, not passive.  Taboo talking.    First impressions count. |  | This study identifies important areas that contribute to the successful cultivation of positive therapeutic alliance. |
| Smith et al., (2016) | What are the experiences of women currently undergoing specialist inpatient treatment for AN? | Lived experience of  inpatient treatment:  Experiencing a process of change.  Coping and adjustment to change & recovery. | Shifts in control.  Handing over of control of eating behaviours.  Living within the restrictive environment.  Regaining control.  Seeking greater collaboration in treatment.  Experience of transition.  At the start I didn’t want to be here.  Treatment as a safe environment.  Loss of normality: Inside the safety bubble.  Back to reality: Concerns of loss of support and fear of relapse.  Process of recovery and self-discovery.  Recovery as an arduous process: Contrast between physical restoration and AN recovery.  Self-discovery and regaining confidence.  Sharing with peers.  I am not alone: Benefits of peer support.  Learning from peers.  Envy of emaciated peers.  Coping with others distress.  Importance of supportive staff relationships.  Trust and making connections.  Being seen as whole person not a walking eating disorder.  Perceived difficulties with staff support. | Findings suggest that patients experience a process of change and adjustment in relation to levels of perceived personal control, attachment to the treatment environment and a sense of self-identity. |
| Stockford et al., (2018) | What are women’s experiences of Severe and Enduring AN (SE-AN) and its treatment? | Understanding of the development of AN  Experience of AN as functional – A solution to other problems  Negative effects of AN  Lack of early intervention  Cycle of accessing services  Negative experiences of staff attitudes  Experience of being with other patients in specialist services | Specific Trigger  Perfectionist Personality  Chronic low self esteem/worth  Distraction to cope/survive  Safety in predictability  Focus away from a lack of valued roles  Who am I? Anorexia identity  Negative impact on life  Lack of understanding from family/friends  Social life  Impact on family  Feelings of hopelessness  Family denial  Waiting lists  Feeling desperate for help  Feeling overwhelmed by food/weight gain  Transitions  Focus on food, weight restoration versus psychological understanding  Treated as a group of patients  Feeling neglected/judged vs being accepted/cared for  Support/friendships  Competitiveness/comparisons  Impact of others distress | Findings suggest that AN functions to resolve complex psychological difficulties including a diminished sense of self worth and a search for identity and that unhelpful treatment experiences and negative relationships have a role in the maintenance of AN. The findings are discussed in the context of a need for services to move away from focusing on eating and weight to assist people with SE-AN to address underlying psychological issues. |
| Wright and Hacking (2012) | Exploration of the therapeutic relationship among adults who access day care services for AN and their healthcare professionals. | Authenticity of the relationship.  Safety  Externalization of the eating disorder.  Recovery measured in kilos  The power of hope and optimism and materialism. |  | Findings suggest that patients appreciated the safety and security of care, but some were using the service as respite rather than recovery. Patients saw goals and tasks related directly to weight gain as irrelevant to their main concerns, but engagement with people who provided a secure, nurturing and maternalistic context for safety and optimism was seen as supportive. |
| Zainal et al., (2016) | Evaluate patients’ treatment experiences of MANTRA and SSCM. | MANTRA.  SSCM.  Non-specific. | Treatment aspects.  Use of manual.  Treatment focus.  Treatment outcomes and recovery.  Positive outcomes.  Effect on understanding AN symptoms and their impact on behaviour.  Effect on feelings and thought processes.  Effect on eating habits and weight.  Effect on communication.  Negative outcomes.  Stages of recovery.  Treatment aspects.  Lack of structure.  Focus on Nutrition.  Treatment outcomes and recovery.  Positive outcomes.  Effect on understanding AN symptoms and their impact on behaviour.  Effect on feelings and thought processes.  Effect on eating habits and weight.  Effect on communication.  Negative outcomes.  Stage of recovery.  Duration, frequency and disruptions of therapy.  External social support.  Pacing and individualization of treatment.  Therapist.  Concerns about ending therapy. | This study suggests that patients experienced the two treatments differently in terms of characteristics and outcomes. MANTRA patients were more willing to express their views on treatment and generally felt more positively about this than those receiving SSCM. |

Table C: Exemplar data extracts for Meta-theme 1: Grappling with Identity

| Sub-theme | Illustrations | Comments |
| --- | --- | --- |
|  | ***EXTRACTS 1:*** *My head teacher, head of year phoned my parents because I had fainted quite a few times and he expressed his concerns to them and they just kind of shouted at me really. They didn't really… they thought it was a phase. They didn't really understand it and threatened to take me to the doctors but they never did. (Sarah, Stockford, 2018, p. 134)*  *He was like ‘Oh you can’t be that bad because you’re not throwing up in bags, you’re not hiding it’, … by the time I finished with him I was throwing up all day every day.* ([Sarah, Rance et al., 2017, p. 589](#_ENREF_39)).  *I think it's kind of a BMI thing is quite deceptive in terms of it's use by medical professions in terms of determining who needs help and therefore it contributes to the anorexic thinking that ‘oh well I'm not actually ill enough I don't actually deserve the help, I need to go out and be more anorexic to get more deserving and achieve more whatever it is’* *(*[*Helena in Stockford et al., 2018, p. 135*](#_ENREF_46)*)*  **EXTRACT 2:** I was sick for a long time before I realized that myself […] In the end, I couldn`t cope with it anymore. I just broke into pieces . ([Mary, in Gulliksen et al., 2015, p. 212](#_ENREF_21))  **EXTRACTS 3:** [The treatment] was great in helping me to see it as a disease with symptoms as opposed to something wrong with me as a person. ([P16, in Zainal et al., 2016, p. 6](#_ENREF_56))  I wasn’t listened to. I was not my name and I felt wiped out. I was not me, I was the eating disorder. That was tough. I felt like I had lost myself already by being sick, […] I had no identity left. ([Cecilia, in Gulliksen et al., 2012, p. 936](#_ENREF_20)).  **EXTRACTS 4:** I felt that it (AN) was something I had chosen myself. Because it wasn’t a chronic disease or anything, like cancer or things like that ([Alicia, in Gulliksen et al., 2015, p. 211](#_ENREF_21)). It’s my own fault and that makes it worse … I’m the problem. ([Denise, in Hannon et al., 2017, p. 288](#_ENREF_22)) […] on the one hand, I’m not thin enough/don’t starve myself […] enough to be anorexic.... on the other hand, you say, well, there’s no way I’m doing this, like, on purpose… and, after all, I was hospitalized twice […] so there’s probably something there. And then you say, alright, maybe I am. ([Emily, in Eli, 2014, p. 4](#_ENREF_12)) I’m not worthy of this title, I didn’t spend enough on it… not enough blood, not enough tears, not enough suffering ([Vered, in Eli, 2014, p. 4](#_ENREF_12))  **EXTRACTS 5**: When I was so ill I felt like I was two people. I’d got the anorexia and I’d got me, and I was really confused, and it was a battle […] I know who Helen is but where does the anorexia and the negativity fit? I know it’s there but where does it fit in to make the whole person? ([Helen, in Ross & Green, 2011, pp. 114-115](#_ENREF_41))  Although I was scared and worried about losing my eating disorder, I also wanted to get into treatment. […]. Because I was so troubled. I wanted to get more meaningful days, to feel better about myself. ([Irene, in Gulliksen et al., 2015, p. 214](#_ENREF_21))  **EXTRACTS 6:** I find it difficult to distinguish … what is me and what is the eating disorder … a lot of what my treatment has been is actually finding my own identity ([Participant 3, Smith et al., 2016, p. 23](#_ENREF_45))  […] it is assumed that every single thing we say is an eating disorder. Yes, sometimes it is but people genuinely do have likes and dislikes.([Participant 11, Smith et al., 2016, p. 22](#_ENREF_45)) | Struggles to locate the AN experience as problematic was systemically (albeit inadvertently) reproduced for some a participants by families who did not acknowledge the seriousness of their child’s struggles with AN.  The seriousness by which health care providers understood the person’s experience had profound implications for participants, with these participants being recruited into increasing the intensity of ED behaviors in order for their distress to be validated and positioned as deserving of treatment.  This struggle to be validated paralleled an internal struggle to discern the experience as problematic that included navigating the question of whether or not the person saw themselves as “sick”.  For some participants, conceptualizing their experience as a “disease” and “an illness” was preferred over alternative conceptualizations (e.g. “something wrong with me as a person”).  For others, an ED diagnosis, particularly when experienced as a “textbook” approach ([Rance et al., 2017, p. 589](#_ENREF_39)), for some participants such as Cecilia, also contributed to a sense of loss of identity.  When participants experienced choice in the development of AN, this disqualified them from an illness or disease discourse that assumes an absence of personal agency and contributed to self-blame (“my fault”).  The question of whether or not a participant qualified in the location of their experience as AN hinged on questions including (1) whether they experienced themselves as agentic in the development of AN; (2) whether they identified as having invested enough of themselves into AN; and (3) the extent of their suffering.  This extract exemplifies how a number of participants were active in negotiating with the question of whether the ED was part of them or not with some participants drawing on an adversarial metaphor (e.g. “battle”) to capture the confusion that was experienced with the sense of a fractured self with different, incoherent parts.  Although AN was frequently internalised, participants who located the AN experience as part of themselves were also frequently “troubled” by this.  Central to the treatment experience for this participant was a discernment and clarification of their identity.  Yet others risked being silenced, losing their identity through being externalized with AN ([Conti, Calder, Cibralic, Meade, & Hewson, 2017](#_ENREF_8)) when it was assumed by others that “every single thing we say is an eating disorder”. |

Table D: Exemplar data extracts for Meta-theme 2: Negotiating Therapeutic intervention

| Sub-theme | Illustrations | Comments |
| --- | --- | --- |
| 1. Therapeutic focus and timing | ***EXTRACTS 1:*** *There is something really good about the fact that there’s an organized diet, that there’s some sort of certainty […] And it spares you this engagement, a certain part of the engagement that used to exist at an obsessive level.* ([Alon, in Eli, 2014, p. 6](#_ENREF_11))  *The second hospitalization, in comparison, was very traumatic. I felt really bad there. I couldn’t find myself… Being in a closed ward with very tough discipline, very clear rules, where they decide for you when you’ll eat, when you’ll have time for breaks, like – it didn’t suit me anymore. I needed my freedom, to decide on my own structure.* ([Meital, in Eli, 2014, p. 7](#_ENREF_11))  **EXTRACTS 2:** [I] spent two years with them [local ED service] … to get to the point where I realized that focusing on food and maintaining restoring weight doesn’t work for me, because all it does is push me further into depression … which I don’t handle very well … it makes me suicidal ([Megan, Rance et al., 2017, pp. 587-588](#_ENREF_38)) You feel very alone, you put weight on and then you’re told you can go when you’re struggling the most with your weight. Then you’re on your own, scared, afraid of being a woman, afraid of assumptions being made by others that you’re OK. ([Joanna, in Ross & Green, 2011, p. 114](#_ENREF_40)) | For this male participant, structure and certainty around eating helped him to step back from obsessiveness.  On the other hand, behaviorally focused interventions were troubled for a number of participants, and for some experienced as traumatic including through a loss of self in a structure that lacked transparency and took away control.  Focusing on ED behavior and weight restoration obscured issues entangled with the ED experience for some participants. and most seriously for ‘Megan’ contributed to a loss of hope and increased suicidality.  When the physical crisis was over the psychological crisis re-emerged at which time support for some participants diminished. |
| 1. Personal preferences and participatory action | **EXTRACTS 3:** I think that one to ones we have with our therapists are very important because they help to tackle individuals’ problems. They personalize it for you which I think is necessary. ([Participant 5, Smith et al., 2016, p. 24](#_ENREF_44)) […] Give and take […] I told them what I found useful in challenging [eating disordered behaviors], I told them what I didn’t ﬁnd useful, and they’d do the same—like what they expected of me and that, it was good ([Sly et al., 2014, p. 238](#_ENREF_43))  **EXTRACTS 4:** […] you have been talking to a professional, someone who is supposed to be skilled in these problems. When that didn’t work out, I thought; there is no hope for you. Straight out. This is not working out. I wasn’t able to understand the connection between what he was talking about and my everyday life. The things that I thought were important and wanted to talk about, these things didn’t seem to be of any importance. ([Diane, in Gulliksen et al., 2015, p. 216](#_ENREF_20)) It felt quite rigid and it was like ‘If you understand you have your thoughts and your feelings are reflecting [them] and challenging your negative thoughts … then you will get better, and if you’re not getting better you’re just not trying hard enough’ ([Claire, Rance et al., 2017, p. 588](#_ENREF_38))  **EXTRACTS 5:** But when it’s their job, they should have been able to do something more than sit there nodding, small-talking, and pampering you all the time. Challenge you, give you a kick in the pants ([Erika, in Gulliksen et al., 2012, p. 938](#_ENREF_19))  Yes. I think that in other places I have been, you get pampered too much, like a little sparrow. People care for you because you are so small and tiny. Poor you, you are so thin and so on. That only maintains the eating disorder; you want to remain like that. ([Karen, in Gulliksen et al., 2012, pp. 937-938](#_ENREF_19))  It’s good that someone challenges you to do things that you might not always feel capable of handling. And then to be there for me...also when things get really hard. (Johanna; Gulliksen et. al., 2012, p. 937)  At ﬁrst I was like scared, because they were like telling me stuff I didn’t want to hear, but now, there’s a kind of safety in it, in knowing that no matter what, I can’t get away with stuff I would have in the past. ([Sly et al., 2014, p. 239](#_ENREF_43)) | Tailoring of treatments to the person relies implicitly on the person having voice to negotiate the therapy focus (i.e. empowering) Alongside having a voice in therapy was a parallel process for this participant in terms of what was expected of her.  The disconnect between the therapist focus on this participant’s life and failure to address what was relevant to them eroded a sense of hope in themselves and their ED recovery.  The therapist assumption that the cognitive intervention of challenging negative thoughts when rigidly applied leads to recovery meant that in the absence of this, this participant experienced blame being allocated to her for the absence of change.  Extracts 5 illustrates a spectrum from wanting treatment and to be challenged at the person’s pace. Central to this is the question of how therapists discern who needs what and when in terms of self/other and control/agency.  These extract makes explicit what may be absent but implicit ([White, 2000](#_ENREF_53)) in a therapist’s challenge – that was, the person was “capable” of change. |
| 1. Therapeutic alliance and being treated as a person | **EXTRACTS 6:** I was getting to know the people more, I was learning to trust them more and to me I could let them in. ([Helen, in Ross & Green, 2011, p. 115](#_ENREF_40))  You build up trust … you know you can say things to them, and they understand a bit more because they know more about your past. ([Participant 2, Smith et al., 2016, p. 22](#_ENREF_44))  She radiates a lot of self-conﬁdence and is very sure of herself. And when she is sure of herself, then I get to be sure of her as well. Because I am very unsure of myself, and often feel very insecure. ([Anne, in Gulliksen et al., 2012, p. 938](#_ENREF_19))  **EXTRACTS 7a:** […] this therapist is that he treats me like someone, even though he sees that I have a disturbance, he treats me like a person who is able to achieve things in life, who can manage to get rid of this. ([Ava, in Gulliksen et al., 2012, p. 937](#_ENREF_19))  She doesn’t want to work on the eating disorder she wants to work on me … she sees me as a whole person’. ([Amy, Rance et al., 2017, p. 589](#_ENREF_38)).  *[…] to have people that care for you and accept you and that don't look down their nose at you and ya know see you as a worthy human being that's an individual and that can spend time with you not just go shovel you full of cheese and fattening foods with no humanity (Emma, in* [*Stockford et al., 2018, pp. 135-136*](#_ENREF_46)*).*  **EXTRACTS 7b:** […] They didn’t believe me until they found me, fainted.... That’s what annoys me, that they don’t trust you. ([Danielle, in Eli, 2014, p. 5](#_ENREF_11))  … just like, I suppose that I felt like they had given up on me so I gave up on getting involved, just like I can’t be changed so what’s the point really. I feel sad now looking back and just thinking I wish I had the knowledge that comes with age and experience. ([Betty, in Fox & Diab, 2015, p. 33](#_ENREF_16))  **EXTRACTS 8:** Looking at where I am now, you know, I look back to those times and wonder how I got here! It was just so hard you know to keep going, but I think she played such a strong part, really stable, and we just kept going, a bit at a time until, well, here. ([Sly et al., 2014, p. 240](#_ENREF_43))  Yeah – cos you’re a unit and you can battle it together, if Gail says it will be ok – then it will be ok. You can battle together...she always makes you feel like you are in it together. ([Catherine, in Wright & Hacking, 2012, p. 112](#_ENREF_54))  . . . she’s like a little support angel on your shoulder . . . she just makes you feel safe somehow. She makes you feel like she can hold you and the disorder, and no matter what happens she’s got hold of you and don’t worry ‘cos there is somebody there. ([Catherine, in Wright & Hacking, 2012, p. 111](#_ENREF_54))  **EXTRACTS 9a:** My vision is so tunneled I need them [the workers] to help me see the wider zone. I can’t do that on my own cos I am too focused on me...me...me; they give me eyes. ([Lynne, in Wright & Hacking, 2012, p. 112](#_ENREF_54))  They knew about me much more than I knew about myself… things that even I wasn’t aware of, but that they could see from the outside.... It always gave me a good feeling – that I don’t have to talk and they still know . ([Grace, in Eli, 2014, p. 4](#_ENREF_11))  **EXTRACTS 9b:** […] but the punitive thing… that just fuels the disease, that does nothing helpful ([Darcy et al., 2010, p. 267](#_ENREF_8))  All those times I never felt any compassion or heartfelt concern. I can still feel those looks directed at me—looks that tell you that you have yourself to blame for this. ([Ingrid, in Gulliksen et al., 2012, p. 936](#_ENREF_19)) | Trust was an essential component of letting people “in”, implicit within this is a trust that the therapists demonstrated capacity in being able to stand with the person including the disclosure of past (and for some, painful/shameful) experiences.  Trust in the therapist’s expertise was also highlighted as significant. This expertise included both knowledge of ED and confidence that had the effect of rebuilding this participants’ confidence in themselves.  These participants highlighted the significance of being seen and treated as (1) a person whose identity was not confined to the identity of a sick person; and (2) a person who is worthy.  The absence of therapist trust in the person was resisted by participants. Implicit in this absence of trust in the person was that they were incapable of change that Betty repositioned herself on over time.  This extract highlights some of the ways that the participants spoke of being held as a person in “strong” and “stable” (Sly et. al., 2014) ways by their therapist as they sustained steps towards change.  How participants constructed this task for change varied within and between their narratives and was shaped by the metaphors they selected to understand their experience – for example, Catherine drew on a range of metaphors ranging from adversarial (“battle together”) to externalization of identity from the AN through a relational metaphor (“she can hold you and the disorder”).  Within these therapeutic spaces, Lynne experienced an expansion of her vision of herself: “they give me eyes” and for Grace, a therapist looking in from the “outside” generated a sense of comprehensive understanding to reveal senses of herself that had previously been obscured.  The absence of therapist holding in ED treatments was experienced by these participants as punitive and contributed to a sense of blame and erosion of a sense of identity that had the effect of perpetuating AN itself. |

Table E: Exemplar data extracts for Meta-theme 4: Recovering and rebuilding identity

| Subtheme | Illustrations | Comments |
| --- | --- | --- |
|  | **EXTRACTS 1:** I feel that the therapy has given me a much greater understanding of why I keep this illness and what the illness gives me. I strongly believe that I needed this insight to be able to work on recovery. ([P32, in Zainal et al., 2016, p. 6](#_ENREF_55))  He helped me to see that whereas it feels like it’s food, most of the time that what I’m afraid of is life itself. I’m just so frightened of it, because of the hurt that went on in my childhood. [….] I know now that I have to face life that I was so afraid of, that had become so alien and so unkind. I suppose really that’s what I’ve been trying to do. ([Joanna, in Ross & Green, 2011, p. 115](#_ENREF_40))  **EXTRACTS 2**: And he doesn’t have the perspective that ﬁrst we have to get rid of this eating disorder and then you can start your life. It’s more the perspective that it’s possible living with this disturbance and doing things along the way, you know. He puts a lot of faith in that you can get out of a disturbance by starting to live your life differently. ([Ava, in Gulliksen et al., 2012, p. 937](#_ENREF_19))  I want them to give them the same treatment opportunities as people they’ve see for the first time, so to keep believing that recovery is possible for anyone ([Helena, in Stockford et al., 2018, p. 136](#_ENREF_46))  **EXTRACTS 3:** Learning to sit with emotions rather than avoiding them through restricting your food and exercising. ([Eilidh, in Hannon et al., 2017, p. 291](#_ENREF_21))  [Expressing my feelings to others] has been really beneficial as I can tell people when I am struggling, instead of letting the eating disorder do the talking. ([P1, in Zainal et al., 2016, p. 6](#_ENREF_55))  Also understanding how I didn’t need to control food as my way of saying “I’m really annoyed at the moment, I’m really upset”, which was, yeah…the way I used to do it. ([P13, in Lose et al., 2014, p. 134](#_ENREF_28))  **EXTRACTS 4**: I am getting character in myself again and finding a personality and it is all coming back to me who I want to be ([Participant 15, Smith et al., 2016, p. 23](#_ENREF_44)).  Absolutely, deﬁnitely feel a bit more comfortable within myself, you know I feeling very hopeless going back to sort of this time last year […] ([P3, in Lose et al., 2014, p. 134](#_ENREF_28))  But it really did help me find out who I was, all the anger coming out, all the negativity. It was the first time I felt safe expressing that. ([Helen, in Ross & Green, 2011, p. 116](#_ENREF_40)) | Across these participant narratives was the significance of insight in the process of recovery and ways these were generated in the context of therapy. For example, for Joanna, insights included the understanding of AN as a way of understanding and facing emotions and how these are a response to life and history and the reconstruction of life and identity in face of the ED experience and its treatment.  Ava found comfort in her therapist taking up the perspective that the priority is to reclaim life rather than vanquishing the ED from her life. This relational, rather than adversarial, focus of therapy provided a foundation of hope that she could have a life lived differently. Helena highlighted the importance of a therapists hope that recovery is possible regardless of length and severity of AN,  What was significant for these participants was an opening up, allowing for, and voicing their emotions and in their relationships with others and insight into how AN had been a vehicle for emotional expression.  These participant recovery narratives were (re)constructed with themes that included reclaiming lost identities through lived experience that included clarifying to themselves a valued stance of who they were and wanted to be. Therapy was experienced by some participants as a site for finding a sense of identity and as a “safe” place to express previously troubling emotions. |
