# Supplementary material for: “Finding my own identity”: a qualitative metasynthesis of adult anorexia nervosa treatment experiences
Source: BMC Psychol. 2020 Oct 22;8:110. doi: 10.1186/s40359-020-00476-4 (PMC7583290; doi:10.1186/s40359-020-00476-4)
Supplement: Supplementary file 2 — Additional file 2: Tables C, D & E. Exemplar data extracts for Metathemes 1-3 in the Metasynthesis. [file 40359_2020_476_MOESM2_ESM.docx]

**Clinical Significance of paper: “Finding my own identity”: A Qualitative Metasynthesis of Adult Anorexia Nervosa Treatment Experiences**

The clinical significance of this metasynthesis is in highlighting therapeutic challenges and clinical implications in the treatment of adult anorexia nervosa.

1. **Key Therapeutic challenges**: including importance of treatment teams:
   1. Addressing individual differences and balancing treatment interventions to prioritise physical safety and assist the person to find their own identity, including a sense of self-worth and that they are more than the AN.
   2. Preserving personal agency and autonomy within a treatment non-negotiable context and the timing of interventions.
2. **Clinical applications and considerations**: including the importance of therapists and treatment teams:
   1. Checking in with the person within a treatment non-negotiable context that prioritises safety;
   2. Establishing a respectful therapeutic alliance that includes being held emotionally, treated as a person who has a range of self-capacities and is more than the AN, and instils hope; and
   3. Journeying alongside the person in their recovery, including engaging in what recovery means to the person and addressing processes of rebuilding identity.
